# An initial assessment of linkage disequilibrium (LD) in coffee trees: LD patterns in groups of *Coffea canephora* Pierre using microsatellite analysis

**DOI:** 10.1186/1471-2164-14-10

**Published:** 2013-01-16

**Authors:** Philippe Cubry, Fabien de Bellis, Komlan Avia, Sophie Bouchet, David Pot, Magali Dufour, Hyacinthe Legnate, Thierry Leroy

**Affiliations:** 1 CIRAD, UMR AGAP, Montpellier, F-34398, France; 2 Department of Biology, University of Oulu, PO Box 3000, Oulu, FI-90014, Finland; 3 INRA, UMR de Génétique Végétale, INRA/CNRS/Univ Paris-Sud/AgroParistech, Ferme du Moulon, Gif sur Yvette, F-91190, France; 4 CIRAD, UMR CMAEE, Montpellier, F-34398, France; 5 CNRA, Divo, BP 808, Côte d’Ivoire; 6 Current address: DBN Plant Molecular Laboratory, National Botanic Gardens of Ireland, Dublin, Glasnevin, Dublin 9, Ireland

**Keywords:** Africa, Association studies, *Coffea canephora*, Genetic diversity, Linkage disequilibrium, Microsatellite

## Abstract

**Background:**

A reciprocal recurrent selection program has been under way for the *Coffea canephora* coffee tree for approximately thirty years in the Ivory Coast. Association genetics would help to speed up this program by more rapidly selecting zones of interest in the genome. However, prior to any such studies, the linkage disequilibrium (LD) needs to be assessed between the markers on the genome. These data are essential for guiding association studies.

**Results:**

This article describes the first results of an LD assessment in a coffee tree species. Guinean and Congolese breeding populations of *C. canephora* have been used for this work, with the goal of identifying ways of using these populations in association genetics. We identified changes in the LD along the genome within the different *C. canephora* diversity groups. In the different diversity groups studied, the LD was variable. Some diversity groups displayed disequilibria over long distances (up to 25 cM), whereas others had disequilibria not exceeding 1 cM. We also discovered a fine structure within the Guinean group.

**Conclusions:**

Given these results, association studies can be used within the species *C. canephora*. The coffee recurrent selection scheme being implemented in the Ivory Coast can thus be optimized. Lastly, our results could be used to improve *C. arabica* because one of its parents is closely related to *C. canephora*.

## Background

### Introduction

Plant breeding is making increasing use of molecular markers to speed up selection cycles and thereby more rapidly introgress interesting zones of the genome in varieties of agronomic interest. In addition to QTL analyses, which only focus on a single progeny, association genetics can be used to focus on a set of populations to identify zones of interest of the genome in breeding. However, for association studies to be successful it is necessary to know the degree of linkage disequilibrium (LD) beforehand, along with its extent and its distribution within the species studied. This disequilibrium, which measures the intensity of linkage between markers, is used to determine the molecular marker density of the genome, which is necessary for association studies to be effective [1-3].

Association studies are particularly relevant in perennial species, as they can be carried out on pre-existing populations, in collections or in selection trials. They do not require the creation of specific populations by controlled crossing, as is the case with conventional genetic mapping approaches [4]. Association studies have proven effective in a large number of plant species, including maize [5] and grapevine [6]. A prior study of the LD in a species guides the choice of one of the two association study possibilities: genome-wide scan or candidate gene approach.

No large-scale LD study has been undertaken yet on the coffee tree. *Coffea canephora* Pierre ex A. Froehner (2n = 2X = 22) is a strictly outcrossing diploid species with a genetic self-incompatibility system [7,8]. It provides 41.3% of the world coffee production [9]. One of the most ambitious genetic improvement programs for this species has been conducted in the Ivory Coast and is based on a reciprocal recurrent selection scheme (RRS) that was launched in the 1990s [10,11]. This selection program uses the genetic diversity of the species by creating hybrids between the genotypes of two genetic groups: Congolese from central Africa and Guinean from western Africa. The combined use of the RRS and association genetics could help to more effectively guide crosses and speed up the introgression of specific alleles identified as being of interest through the early selection of genotypes derived from crosses.

However, natural populations of *C. canephora* are relatively small in size. The strictly outcrossing reproduction system of this species and the different levels of existing kinship create complex genetic structures at the population scale. This structure in populations is superimposed on the larger diversity group (DG) scale [12].

Our work, therefore, consisted in assessing the LD within the Guinean and Congolese DGs of *C. canephora* as identified using molecular markers [12-14].

The expected results were as follows: i) knowledge of the LD dynamics at the genome level for a certain number of coffee diversity groups or populations, ii) enhanced knowledge of the genetic structure of the Guinean diversity group, and iii) identification of populations that can be used in association genetics.

## Methods

### Plant material

We studied 356 genotypes of *C. canephora* divided into five DGs based on the diversity analyses carried out in previous studies [12,13] (see Table 1):

DG G, Guinean diversity group: a mixture of different wild or cultivated genotypes collected in the Ivory Coast and Guinea. It includes the populations Mouniandougou, Ira 1, Ira 2, Fourougbankoro, Piné, and cultivated Guinean clones

DG GP, Pélézi diversity group: a natural Guinean population related to the Guinean diversity group, isolated from a forest of the Ivory Coast

DG C, Nana diversity group: a population collected in the Central African Republic (CAR)

DG SG2: genotypes cultivated in the Ivory Coast and Uganda or in collections in Brazil, most likely originating from the Congo basin

DG SG1: genotypes cultivated in Togo and Benin (Niaouli population) or from surveys in the Luki collection (Democratic Republic of Congo, Luki population), originating from the Atlantic seaboard, from Gabon to the Democratic Republic of Congo

**Table 1:** **Characteristics and origins of the*C****.****canephora*diversity groups**

**Name**	**Origin**	**Diversity group**	**Size**	**Type**
Nana	Central African Republic	C	92	Spontaneous
Pélézi	Ivory Coast	GP	35	Spontaneous
Guinean	Ivory Coast – Guinea	G	128	Cultivated & spontaneous
SG1 Bulk	Atlantic Coast (Gabon – Congo – Democratic Republic of Congo)	SG1	16	Cultivated & spontaneous
SG2 Bulk	Congo basin	SG2	85	Cultivated

### Choice of microsatellite markers and genotyping

**Figure 1:**
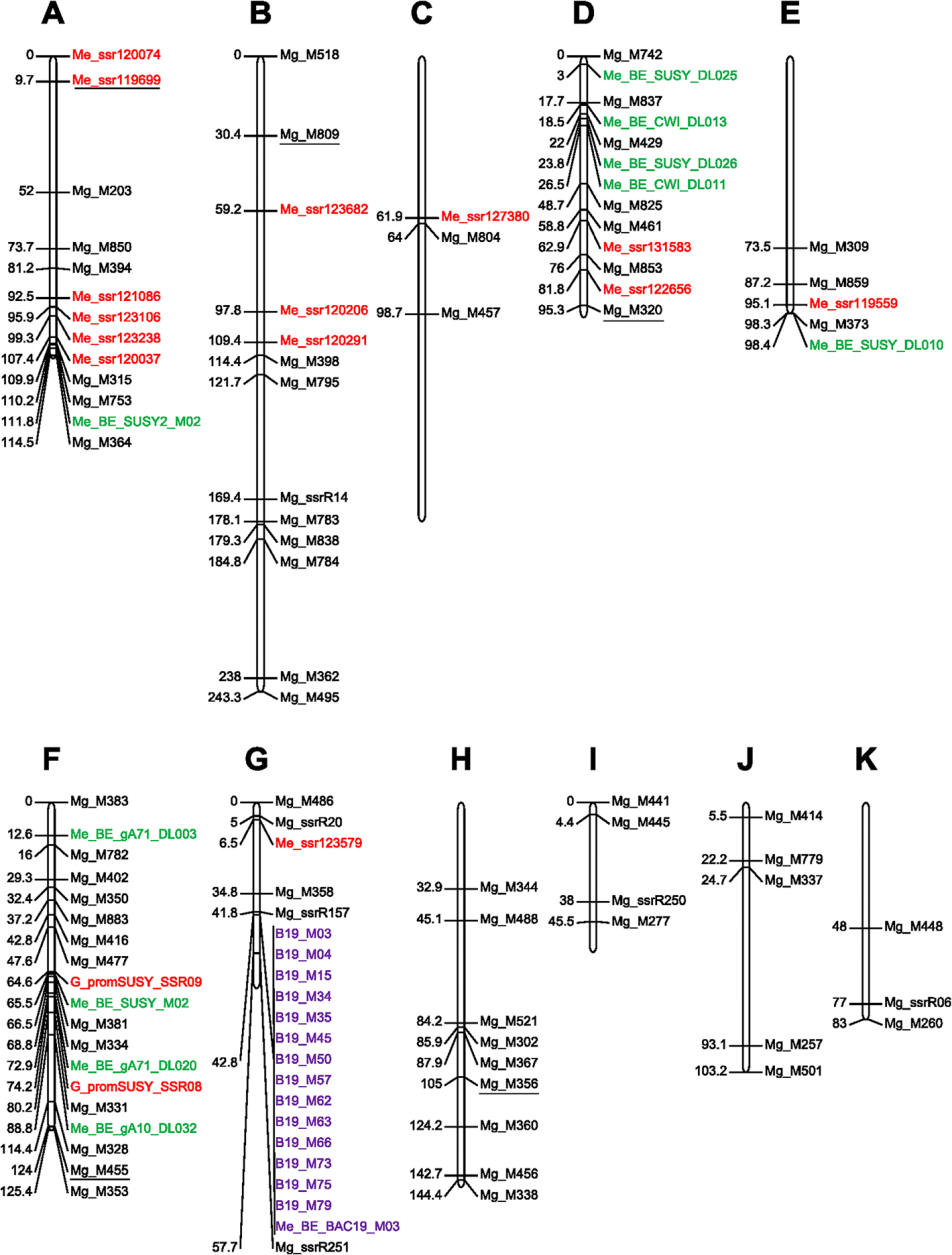
**Location on the genetic map of the 108 microsatellite markers used for the linkage disequilibrium study.** In black, markers from genome banks; in red, markers derived from EST or gene sequences; in green, markers developed on sequences of BAC-ends; and in purple, markers developed on sequences of BAC 111O18. Markers not present on the genetic map published by Leroy et al. (2011) are underlined.

All individuals were genotyped using 108 microsatellite markers mapped to eleven linkage groups (LG, called A to K) on a *C. canephora*[15] linkage map that spanned a length of 1320 cM (Figure 1). The markers are described in Additional file 1: Table S1.

All LGs were studied with a larger number of markers on LGs A, B, D, F, G and H. The average distance between markers was 13 cM, ranging from 0 cM for the closest marker pairs to 243.3 cM for the most distant marker pairs.

The genotyping was performed according to the protocol described in [16]. Size controls were replicated on the different gels to ensure the uniformity of genotyping data. Data were imported into the Microsoft EXCEL® spreadsheet from SAGA GT® (LI-COR Biosciences, Lincoln, Nebraska, USA) and were formatted for the different data analysis software used.

### Genetic structure validation of the sample

A model-based Bayesian analysis implemented in STRUCTURE [17,18] was performed to validate the DG structure of our sample. We ran a correlated-allele model with 10,000 iterations and 10,000 burn-ins; ten runs were made for each assumed K (putative number of populations), with K varying from one to ten. The ad-hoc statistics Δ(K) proposed by [19] were used to assess the number of populations. The Hardy-Weinberg Equilibrium (HWE) and summary statistics, including the number of alleles and the expected and observed heterozygosity for each locus, were computed for the entire sample and for each DG using ARLEQUIN software v. 3.5.1.2 [20]. We also computed the AMOVA and F-statistics for diverse levels of population structure using ARLEQUIN.

### Genetic structure of diversity group G

The Guinean diversity group is composed of a large number of natural populations from the forests of the Ivory Coast, including the Pélézi population (DG GP, see above), which exhibits some original characteristics. The wide variety of these populations, as well as the existence of Guinean genotypes taken from smallholder plantations in the living collections in the Ivory Coast, further justify an in-depth study of this diversity group.

Earlier diversity studies did not offer sufficient resolution to analyze the genetic structure of this group in a satisfactory manner (lack of markers). The genetic structure of this group was analyzed using DARwin software based on the calculation of genetic dissimilarities between individuals followed by a factorial analysis from a dissimilarity matrix (FADM) and the construction of a neighbor-joining tree (NJ) [21,22]. For comparison purposes, we ran a STRUCTURE analysis within the DG G using the same parameters and procedure as above.

### Statistical data analysis and LD analysis

#### Reconstruction of haplotypes

The species *C. canephora* is a highly heterozygous species. Therefore, it is difficult to distinguish the allele phase of the double heterozygotes Aa/Bb, i.e., whether A is associated with B or with b at the haplotype level [23]. However, the most common and powerful measurements of the LD (D, D’, r^2^) rely on an estimation of the haplotypic or gametic linkage disequilibrium [24], i.e., using the allele phase information at the gamete level. Consequently, to estimate these measurements, it is necessary to have access to the haplotypes. Therefore, we used PHASE software [25-27] to reconstruct the haplotypes. This software estimates the most probable haplotypes for each genotype based on an EM algorithm (Expectation-Maximization) that incorporates a coalescence hypothesis in a maximum likelihood model. The haplotypes are reconstructed following a certain number of strong hypotheses using parameters such as allele frequencies, the possibilities of recombination between markers and simulated allele pedigrees.

We used the entire set of 356 genotypes, as the algorithm functions better with genetically structured data [27]. Nevertheless, the dataset was partitioned by the LG of the genetic map (11 matrices in all), as the markers situated on different LG could not be in phase. This approach has been shown to be effective, and the gain in power has been proven, as in the case of grapevine [28,29].

To enable the use of PHASE while maintaining the Stepwise Mutation hypothesis for the microsatellite markers, our data that were expressed in allele sizes were converted into repeat numbers using CREATE software [30].

Five algorithm repeats based on 1000 iterations, 100 thinning intervals and 1000 burn-ins were performed, and the repeat displaying the greatest maximum likelihood was used for the remaining analyses.

The resulting tables adopted for each LG were then merged and formatted for incorporation into PowerMarker to analyze the LD, declaring genotype data of the known phase as the data type.

#### LD analysis

The LD was analyzed for the five DGs, together and separately. We calculated the *D’* and r^2^ values for the set of possible combinations of markers, two-by-two, using PowerMarker software [31]. These measurements were initially developed for bi-allelic loci. Nevertheless, an estimation of these measurements for multi-allelic loci can be performed by establishing a weighted mean for the set of disequilibria between allele pairs [32,33].

Exact Fisher tests were carried out for all the possible combinations to test whether the haplotype frequencies between two loci were the product of the allele frequency corresponding to the two loci. Allele counting was organized in a contingency table, and permutations, following an algorithm using a Markov-Monte-Carlo chain, were used to calculate the unbiased p-values associated with the test [33]. We corrected the significance limit of the p-values associated with the exact test using Bonferroni’s correction to more effectively overcome the effect of the very large number of tests performed. We graphically represented the r^2^ values as a function of distance for each of the groups studied.

## Results

### Genetic structure of the entire sample

**Figure 2:**
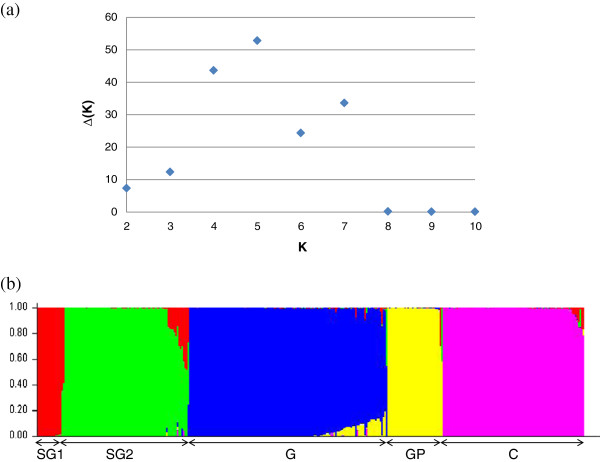
**(a) Ad**-**hoc statistics** Δ**(K) based on STRUCTURE lnP(D) summarized over 10 reps for each K (assumed number of populations) exhibiting a signal at K=5 populations and (b) bar-plot of the STRUCTURE run exhibiting the highest lnP(D) for K=5.** STRUCTURE analysis was performed over the 356 genotypes.

The ad-hoc statistics Δ(K) indicate an uppermost level of structure in five populations. The Δ(K) and bar-plot from the STRUCTURE run showing the highest lnP(D) for K=5 are presented in Figure 2. The expected and observed heterozygosity and Hardy-Weinberg exact test associated p-values for each locus are provided in Additional file 2: Table S2. At the level of the entire population, all HWE departure tests are significant, indicating a high level of structure. Even at the population level (GP), we still detect a significant departure from HWE for a high proportion (56%) of the polymorphic loci. The AMOVA and F-statistics also indicate a high structure in our sample, with more than 38% of the variance due to the DG and with all the *Fst* between DG significant at a 5% level.

#### Genetic structure of diversity group G

**Figure 3:**
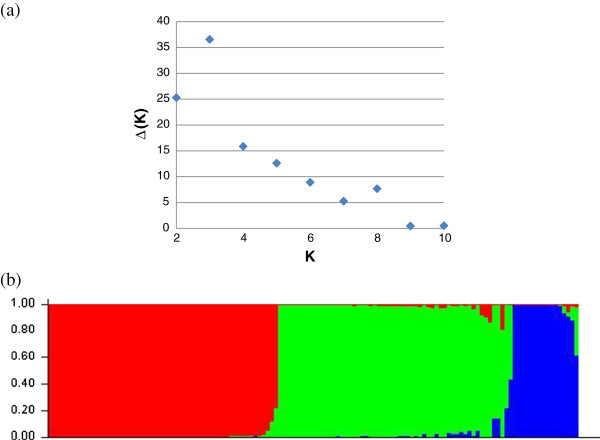
**(a) Ad-hoc statistics** Δ**(K) based on STRUCTURE lnP(D) summarized over 10 reps for each K (assumed number of populations) exhibiting a signal at K=3 populations and (b) bar-plot of the STRUCTURE run exhibiting the highest lnP(D) for K=3.** STRUCTURE analysis was performed over the DG G only.

**Figure 4:**
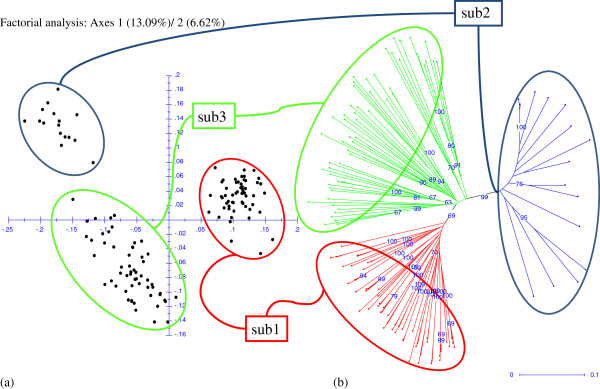
**(a) Genetic structure of diversity group G: first two axes of the associated factorial analysis from a dissimilarity matrix and (b) corresponding NJ tree.** The percentages of total variability are given for each axis. Some coherent subgroups have been identified. For the NJ tree, bootstrap values obtained from 5000 iterations of bootstrap procedure are indicated. For clarity, only values greater than 60 are printed. Unit colors are based on STRUCTURE assignment for each genotype. Probabilities of ancestry greater than 0.6 were considered.

The ad-hoc statistics Δ(K) based on the STRUCTURE analysis gave an uppermost level of structure within DG G in three populations. The Δ(K) and bar-plot from the STRUCTURE run showing the highest lnP(D) for K=3 are presented in Figure 3. The factorial analysis based on dissimilarity index and the NJ tree are shown in Figure 4. Both analyses revealed a marginal structure in three clusters. On the tree, these three clusters corresponded to the Mouniandougou population plus a few individuals from the Piné and cultivated Guinean populations (Gsub1); to the majority of the Ira2 population (Gsub2); and to the Fourougbankoro and Ira1 populations plus a few cultivated Guinean and Piné individuals (Gsub3). For the rest of our work, we considered DG G to be a composite group of different populations, and we also tried to analyze separately the three identified subgroups. The expected and observed heterozygosity and Hardy-Weinberg exact test associated p-values for each locus are provided in Additional file 3: Table S3. The AMOVA analysis and F-statistics of the entire sample, including Gsub1, Gsub2 and Gsub3, are presented in Additional file 4: Table S4.

#### LD analysis at the pan-genomic level

##### LD estimates and their within- and between-linkage group significance by exact Fisher tests and Bonferroni’s correction

Table 2 presents a summary of the exact tests carried out on the different clusters considered. These results demonstrate the importance of the significant associations generated by the genetic structure. In fact, for the set of genotypes, it can be seen that 98% of the marker pairs displayed a significant disequilibrium, regardless of whether they were linked. In addition, DG C and DG GP and the three subgroups of DG G were the only ones to show a within-group: between-group ratio over 1. When trying to consider a finer structure, a certain number of significant between-group associations were no longer detected. For DG G, we found a considerable correction of genomic LD for the set of subgroups (Gsub1, Gsub2 and Gsub3) in relation to DG composite G.

**Table 2:** Analyses of associations between markers per exact Fisher test for the different genetic groups considered

**Diversity group**	**Number of individuals**	**Number of polymorphic markers**	**Number of 2-by-2 tests**	**Limit(5%)after Bonferroni’s correction**	**Number of significant associations(exact test)**		**Number of significant within-linkage group associations and percentage compared to the total significant associations**		**Number of significant between**-**linkage group associations and percentage compared to the total significant associations**		**Within**-**linkage group: Between-linkage group ratio**
All genotypes	356	108	5778	8.65351E-06	5684	98%	680	***12****%*	5004	***88****%*	0.14
C	92	98	4753	1.05197E-05	72	2%	62	***86****%*	10	***14****%*	6.20
SG1	16	93	4278	1.16877E-05	177	4%	29	***16****%*	148	***84****%*	0.20
SG2	85	107	5671	8.81679E-06	389	7%	144	***37****%*	245	***63****%*	0.59
Pélézi	35	74	2701	1.85117E-05	116	4%	85	***73****%*	31	***27****%*	2.74
G	128	97	4656	1.07388E-05	483	10%	99	***20****%*	384	***80****%*	0.26
G sub1	54	85	3570	1.40056E-05	73	2%	49	***67****%*	24	***33****%*	2.04
G sub2	17	73	2628	1.90259E-05	38	1%	32	***84****%*	6	***16****%*	5.33
G sub3	57	96	4560	1.09649E-05	36	1%	20	***56****%*	16	***44***%	1.25

#### Decrease in LD with genetic distance for the set of genotypes

**Figure 5:**
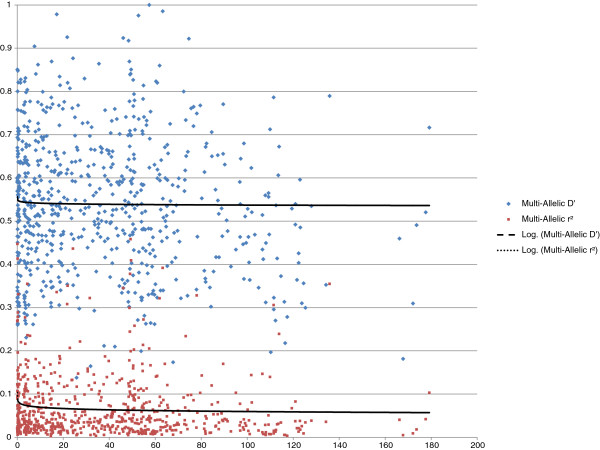
**Decrease in LD (measured by *D’*and r**^**2**^**)as a function of genetic distance in centimorgans for the set of genotypes.** Only significant values after Bonferroni’s correction are shown. The logarithmic regression curves were calculated for all the data.

Figure 5 shows the r^2^ and *D’* values (after Bonferroni’s correction) as a function of distance for the set of genotypes. In general, the *D’* values were much higher than the r^2^ values. In addition, it seems that these values were much more stochastic with large proportions of very high LD that corresponded to non-significant associations. When the different DGs were considered (data not shown), *D’* seemed to be very sensitive to the structure effects and was also sensitive to the variations in allele frequencies between the different markers. Consequently, although the values of the two measurements appeared to decrease with distance, it seems that *D’* was less sensitive than r^2^ to that decrease, with values remaining high at very long distances.

As these two parameters have different properties, the information provided is not redundant. Indeed, *D’* measures only the recombination history, whereas r^2^ measures both the recombination and mutation [34]. These different properties signify that *D’* is globally higher than r^2^ and can potentially reveal more associations. The calculated values of *D’* are lower when population sizes are small, which is why this parameter is preferred when studying the evolutionary history of large populations. However, with association studies, the most indicative value of potential power is the r^2^ measurement, as it provides an indication of the way the markers and phenotypic traits being studied will be correlated. We cite the *D’* for a general comparison on the set of individuals before discussing in detail some differences in r^2^ between the different populations.

#### Decrease in LD with distance for the different diversity groups

The results of this analysis revealed a general decrease in the LD with distance. Nevertheless, we found as many different cases as there were DGs studied.

#### Diversity groups SG1, SG2 and G

**Figure 6:**
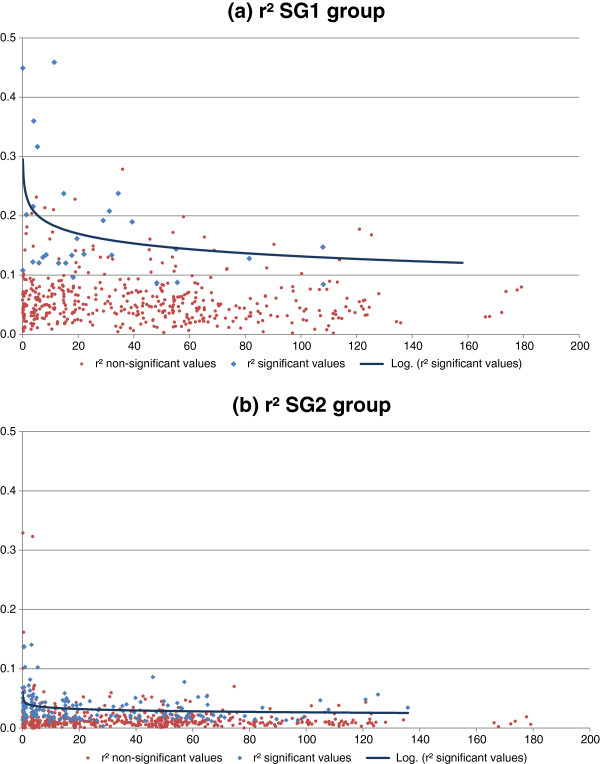
**Decrease in LD (measured by r**^**2**^**)as a function of genetic distance for groups SG1 *(a) *and SG2 *(b)*.** The regressions were calculated on significant values only (blue values).

For DG SG1 (Figure 6), we found moderate to high r^2^ values compared to the other populations, with a large proportion of between-LG LD. The major genetic structure in two populations (Niaouli and Luki) helps to explain such results. In light of these results, it is difficult to choose an r^2^ limit that can be used in association genetics for this DG and that will avoid the risks of false detections.

In DG SG2 (Figure 6), a large share of the significant r^2^ values was found between unlinked markers. The r^2^ values were extremely low, even for some very close markers. This observation may be explained by the origin of this DG, which must have undergone greater genetic mixing than the other origins. Indeed, this group originated from a major center of *Coffea canephora* diversity and is constituted mainly by genotypes resulting from the cultivation and selection processes. It can also be linked to the high number of alleles found in this DG, resulting in a more effective recombination process and diluting the LD signal. For this DG, it is possible to hypothesize that obtaining comparable values between linked and unlinked markers is not due to a structural phenomenon, but rather to very low or non-existent LD in this population. Indeed, structure analyses indicated that this diversity group is not structured in sub-populations and that most of the genotypes within this group are of a cultivated origin. Consequently, more than one microsatellite per cM would be needed in this DG to have any hope of satisfactorily covering the entire genome.

**Figure 7:**
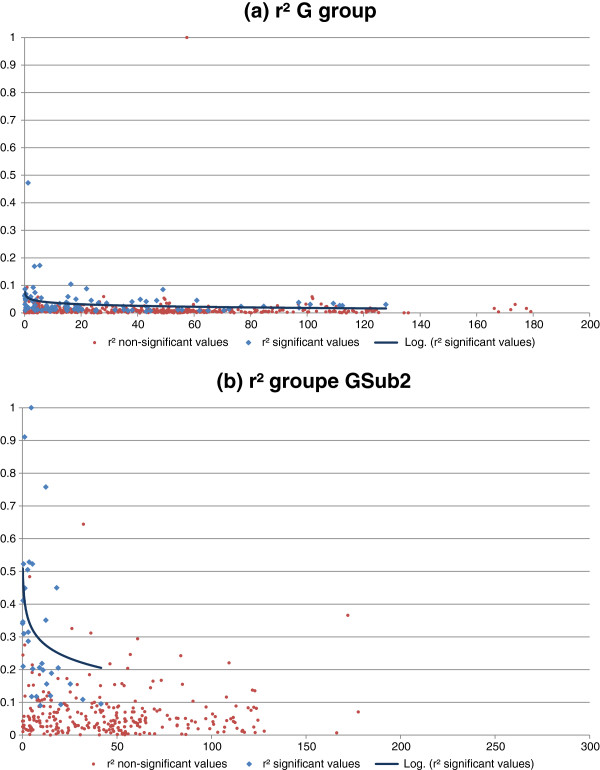
**Decrease in LD (measured by r**^**2**^**)as a function of genetic distance for groups G *(a) *and subgroup Gsub2 *(b)*.** The regressions were calculated on significant values only (blue values).

For DG G (Figure 7), higher r^2^ values between linked markers than between unlinked markers were observed, with a clear decrease in those values with distance. Although some values over 0.1 could be seen for the DG as a whole, this was not the case for subgroups Gsub1 and Gsub2. Indeed, in these two subgroups, we found significant and high r^2^ values between close markers. By choosing an empirical r^2^ limit of 0.2, we arrived at an LD covering approximately 5 cM for subgroup Gsub1 and 20 cM for subgroup Gsub2 (Figure 7); these values suggest that it would be possible to cover the entire genome with a reasonable number of markers, i.e., approximately 290 and 70 markers, respectively.

A very low LD was found for subgroup Gsub3, as well as for DG SG2. The LD remained very low for this group, even at low distances. Therefore, more than 1500 markers would most likely be required to cover the entire genome in this population.

**Figure 8:**
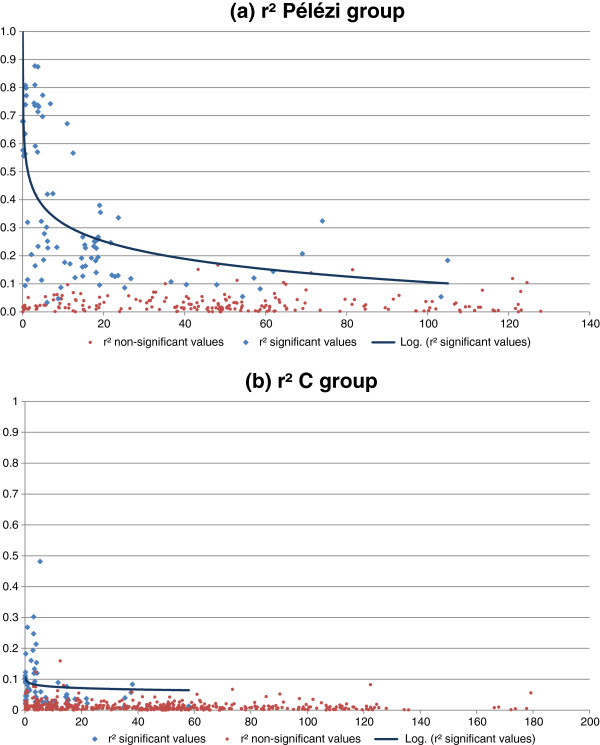
**r**^**2**^**as a function of genetic distance for the Pélézi *(a) *and C *(b) *groups.**

Lastly, DG C and DG GP (Figure 8) exhibited high r^2^ values between close markers, displaying a strong decrease with distance, with a faster decrease in C than in GP. When an r^2^ value limit of 0.2 was chosen for GP and 0.1 was chosen for C, the LD was significant over a distance of approximately 23 cM for GP and 5 cM for C. These distances suggest that association studies can be conducted on these two populations with approximately sixty-five and 290 markers for GP and C, respectively.

Knowledge of the global LD thus provides a general idea of the variations in the LD over the entire genome. However, as the LD is highly variable depending on the region, a separate analysis of the various LGs for DGs GP, C, SG2 and G was carried out to compare the LD depending on the genome region.

#### Comparison of LD patterns between linkage groups

**Figure 9:**
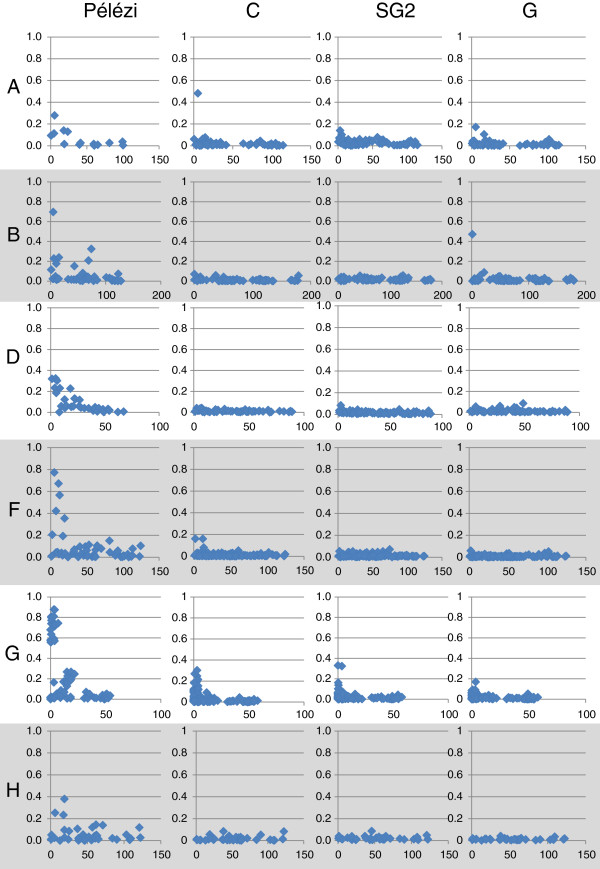
**Decrease in r**^**2 **^**as a function of distance for 6 linkage groups for a few diversity groups.**

The graphs for r^2^ as a function of distance for LGs A, B, D, F, G and H are given in Figure 9 for DGs GP, C, SG2 and G.

The analysis of these results shows that the LD varied depending on the LGs and the DGs that were considered. We confirmed the virtual absence of any usable r^2^ values for DGs SG2 and G. In contrast, for DG GP, we found moderate to high values for the set of LGs, although there were some large differences between the LGs. For DG C, only LGs A, F and G seemed to display r^2^ values over 0.1. However, these results need to be examined further, particularly with regards to LG G, which had a much greater marker density than the other LGs.

When observing the LD matrices per DG (data not shown), significant p-values were preferentially located close to the diagonal, and hence, between linked markers. This preferential localization was particularly apparent for DGs GP and C. In contrast, DGs SG2 and G displayed a large share of significant p-values outside the LGs. DG C only had a few r^2^ values over 0.1, reflecting the short distance at which the LD can be detected. However, DG GP had a larger number of values over 0.1 within the different LGs with a LD that seemed to be organized in blocks.

## Discussion

This LD study is the first of its kind in the genus *Coffea*. We identified numerous different cases of LD evolution along the genome within the different DGs. We were also able to identify a cryptic structure within DG G. This very fine genetic structure had not been discovered in earlier studies either due to a lack of resolution or because too few markers were used.

### What is the point of a genome-wide LD study?

As in all species, we found a decrease in the LD with distance. For a population in equilibrium between mutation and genetic drift, the LD (measured by r^2^) is expected to depend on both the effective size of the population and the recombination rate between the loci considered [23,24]. The closer any two markers are, the longer it will take for the LD to dissipate. Therefore, one expects to find some LD values greater between close markers than between distant markers at a given moment in the evolution of a population.

The approach we adopted, with one marker every 13 cM on average but with highly densified portions of the genome, seemed to be the best strategy for constructing an initial pan-genomic view of the LD properties in the species studied. Moreover, our approach enabled a comparison of the LD behavior over different LGs. This comparison should lead to a clearer understanding of the possibilities for association studies within the studied populations.

### Is LD in *Coffea canephora* an insurmountable problem for association studies?

Our results demonstrate that it is possible to perform association studies by working specifically on each population, but not on the global diversity level, in the species. Depending on the populations, the needed marker density varies, but the prospects of using association studies to support breeding programs for our species are quite interesting.

Indeed, the graph showing the set of r^2^ and *D’* values on the scale of the 356 genotypes, combined with the importance of the genetic structure found in the entire sample, clearly illustrates the importance of the structure effect on the detection of associations between unlinked markers. The Hardy-Weinberg disequilibrium could not be reduced from the whole sample scale to the GD level. However, disequilibrium still exists within natural populations of *C. canephora,* as shown by [12,35]. This disequilibrium thus prevents the implementation of association studies for an entire set of genotypes using simple correlation models, which do not take into account structure and kinship effects. This result was confirmed by the large number of significant correlations between markers located on the different LGs for the 356 genotypes compared to those found on the DGs. Therefore, it seems necessary to work at the population level to more effectively study the LD dynamics in *C. canephora*, as the analyses showed that the most valuable results were obtained on DG GP, DG C and two Guinean subgroups (Gsub1 et Gsub2) corresponding more or less to natural populations.

We were thus able to reveal a high variability in LD within the different DGs, with a large share of residual between-linkage group disequilibrium in DG SG2 and DG SG1. These results may potentially lead to the detection of false positives in association studies, even at low levels of genetic structure. The importance of this “genomic” LD (as opposed to local LD) was variable depending on the groups, and by taking into account structure and kinship in association studies, it will be possible to overcome this variability. The residual genomic LD values for the less-structured DGs may be explained by different kinship levels within the natural populations of our species. Nevertheless, for DG SG2, the low r^2^ values obtained suggest that the LD is significant at very short distances. Indeed, natural populations of coffee trees are usually small, isolated populations with a small number of mother trees and a few juveniles, involving major relations of kinship, despite the strict outcrossing of the studied species.

We used Bonferroni’s correction to consider only truly significant values. Nevertheless, this correction is very conservative and may lead to a substantial loss of power in association studies. Many other corrections have been proposed in the literature in recent years, but none seems to be satisfactory. Moreover, we have shown that, in our case, this correction mainly made it possible to eliminate a certain number of disequilibrium values between unlinked or very weak markers. Normally, in association studies, such a correction will not be necessary because the main source of error (genetic structure) will be controlled. These questions should be given due consideration along with updates in the proposed models. Models that take into account structure and kinship in association studies appear to be a major advance in these approaches, helping to increase both the power and the resolution of such studies [32].

### Genetic structure of DG G

Our study enabled us to more effectively determine a fine genetic structure for DG G. Structure seems to exist in these populations, but there are indications of major gene exchanges between them. We found a structure in three subgroups (Gsub1, Gsub2 and Gsub3) with both model-based and distance-based analyses. This very fine genetic structure can only be studied with a large number of markers. DG G was initially described by Berthaud [36] using isozyme markers. Berthaud concluded at the time that there was an absence of genetic structure within this group. However, Cubry et al. [12,13] showed the existence of a Guinean population that was different from the others (GD GP). It will also be important to study kinships within natural populations, along with the gene flow existing between them, to understand the dynamics of those populations on the forest scale in Guinea and the Ivory Coast.

### What models can be used for association studies in *Coffea canephora*?

Our results show, particularly for the Guineans, that a large number of “control” markers (i.e., control markers that can be used to estimate structure and kinship independently from the association study) are needed to separate the fine structure into populations. Therefore, our case seems to be quite similar to the case of maize, where a set of eighty-nine microsatellite markers was used by Flint-Garcia et al. [37] to study structure and kinship on 302 lines.

After correction of the p-values by the Bonferroni method, some large and significant values of the two LD measurements (*D’* and r^2^) were found both between unlinked and linked markers, preventing any distinction between associations based on a physical link between markers and those created by the structure. Therefore, the genomic control approach (adaptation of the significance limit to the number of associations detected between unlinked markers) appears to be less efficient and may lead to a large number of false negatives. This observation is one of the greatest criticisms of this model advanced by Yu et al. [38]. Moreover, this approach estimates that structure has the same effect at any point of the genome [5].

The structured association approach proposed by Pritchard et al. [17] seems to be more efficient than the genomic control. Nevertheless, the degree of kinship in the populations studied, as shown by the diversity trees obtained (particularly for DG GP), indicates that a share of the confounding effect of genetic structuring is not taken into account in this model. Consequently, it seems that the model best adapted to the species and populations in our study is the mixed model proposed by Yu et al. [38]. This approach has shown its power and its superior control of false positives when compared to other methods using simulated data.

These association study models are becoming increasingly efficient, and we seem to be arriving at a critical point in the development of these approaches. Even so, particular attention must be given to the choice of traits studied and their distribution within the sample on which association studies are performed. Indeed, by correcting the structure effect, there is a risk of not being able to detect traits that would have a distribution superimposed on the population structure [39].

### Which target populations should be used for association studies?

The purpose of our work was to make an initial assessment of the LD at the pan-genomic level in *C. canephora*. We thus discovered considerable variation in the LD between populations. The DGs comprising natural populations, such as GP or C, appear to have a moderate to high LD, at approximately 5 to 25 cM. In these DGs, it seems feasible to carry out genome-wide scan type studies. Nevertheless, given the stochasticity of the LD between LGs and its sensitivity to low allele frequencies, we have certain reservations regarding this type of approach, notably when using highly polymorphic multi-allelic microsatellite markers.

The DGs comprising improved populations, such as SG2, seemed to have undergone substantial genetic mixing with greater diversity and a virtually undetectable LD on the scale at which we worked. Consequently, this type of population seems more suited to regional or candidate gene type approaches.

To conclude, the current association study models can be used to consider structure and kinship effects, enabling this type of approach for use even with composite and structured samples.

### Which approach for association studies in *Coffea canephora*?

For most of the considered DGs, a high density of markers is required to perform association studies. Using SNPs in additions to the SSR should be of great value.

Considering ongoing work on SNP discovery and genotyping by sequencing in coffee, a high number of markers will likely be obtained in the short term. Then, genome-wide association studies (GWAS) can be easily applied to populations used in breeding processes, such as populations involved in the RRS in the Ivory Coast. In countries such as Uganda, GWAS should be applied to the entire germplasm used for breeding for tolerance to Coffee wilt disease (CWD). Therefore, the candidate gene approach should only be used in very low LD populations or for specific purposes.

## Conclusions

We were able to demonstrate a cryptic structure within the Guinean diversity group (DG G). This very fine genetic structure was not detected in earlier studies either due to a lack of resolution or because too few markers were used. Nevertheless, complex dynamics seem to exist within the coffee populations of the Guinean region that are substantially different from those found in the forests of Uganda, as described by Musoli et al. [35]. A more in-depth study of these populations will be undertaken to establish the relationships and gene flow existing between them.

The LD study, the first of its kind in the genus *Coffea*, has enabled us to consider association studies in the species *C. canephora*. In the different DGs, we identified numerous different cases of LD evolution along the genome. This study provided us with a basis for carrying out association studies and thereby optimizing the reciprocal recurrent selection scheme for the genetic improvement of *C. canephora*. This selection scheme is based on the complementary traits of diversity groups and the agronomic value of intergroup hybrids. Association studies within groups will allow us to significantly improve each of the complementary populations for their traits of interest. An initial application of this approach can be employed in Guinean populations (exhibiting high LD) with a limited number of markers throughout the genome. A selection process should be efficient and quick using association studies for the early selection of heritable traits (such as vigor or bean size) in intergroup hybrids.

The major genetic structure of our species, which may have limited the feasibility of association studies a few years ago, can now be taken into account in the latest models. Nevertheless, it will be important to carefully consider all the models and, in particular, to avoid over-correcting the structure effect, as this action may lead to numerous false negatives and a major loss of detection power.

Lastly, the results of our study on *C. canephora* could be extrapolated to *C. arabica.* Although *C. arabica* has a very restricted genetic base, as shown by Lashermes et al. [40] and Anthony et al. [41], one of the species participating in its creation is very close to *C. canephora*.

## Abbreviations

LD: Linkage Disequilibrium; DG: Diversity Group; LG: Linkage Group; cM: Centimorgan.

## Competing interests

The authors declare that they have no competing interests.

## Authors’ contributions

PC, MD, DP, KA, SB and TL designed the study. HL provided the plant material. PC, FDB and KA carried out the genotyping experiments. PC and DP analyzed the data. PC, FDB and TL wrote the manuscript. All authors read and approved the final manuscript.

## Supplementary Material

Additional file 1: Table S1 Detailed information on the 108 SSR markers used. Click here for file

Additional file 2: Table S2 Number of alleles, expected heterozigosity(He), observed heterozigosity and Hardy-Weinberg equilibrium exact test associated p-values (P-value )and standard deviation (s.d.) for each locus and for the whole population and the Diversity Groups SG2, SG1, C, G and GP. NA stands for not available due to monomorphic locus. Click here for file

Additional file 3: Table S3 Number of alelles and Hardy-Weinberg equilibrium statistics for the Diversity Groups G, GP, Gsub1, Gsub2 and Gsub3. NA stands for not available due to monomorphic locus. Click here for file

Additional file 4: Table S4 Amova and F-statistics computation over the 7 DG (SG1, SG2, C, GP, Gsub1, Gsub2 and Gsub3). * : significant value at 5%, ns: non significant value. Click here for file
